# Sphingoid Bases Regulate the Sigma-1 Receptor—Sphingosine and *N*,*N*’-Dimethylsphingosine Are Endogenous Agonists

**DOI:** 10.3390/ijms24043103

**Published:** 2023-02-04

**Authors:** Jing Li, Kenneth A. Satyshur, Lian-Wang Guo, Arnold E. Ruoho

**Affiliations:** 1 Department of Surgery, School of Medicine, University of Virginia, Charlottesville, VA 22908, USA; 2 Small Molecule Screening Facility, Carbone Cancer Center, School of Medicine and Public Health, University of Wisconsin, Madison, WI 53705, USA; 3 Department of Biochemistry and Molecular Genetics, University of Virginia, Charlottesville, VA 22908, USA; 4 Department of Neuroscience, School of Medicine and Public Health, University of Wisconsin, Madison, WI 53705, USA

**Keywords:** sigma-1 receptor, endogenous agonists, sphingosine, *N*,*N*’-dimethylsphingosine, modified native gel approach, molecular docking, ceramide

## Abstract

Both bioactive sphingolipids and Sigma-1 receptor (S1R) chaperones occur ubiquitously in mammalian cell membranes. Endogenous compounds that regulate the S1R are important for controlling S1R responses to cellular stress. Herein, we interrogated the S1R in intact Retinal Pigment Epithelial cells (ARPE-19) with the bioactive sphingoid base, sphingosine (SPH), or the pain-provoking dimethylated SPH derivative, *N*,*N*’-dimethylsphingosine (DMS). As informed by a modified native gel approach, the basal and antagonist (BD-1047)-stabilized S1R oligomers dissociated to protomeric forms in the presence of SPH or DMS (PRE-084 as control). We, thus, posited that SPH and DMS are endogenous S1R agonists. Consistently, in silico docking of SPH and DMS to the S1R protomer showed strong associations with Asp126 and Glu172 in the cupin beta barrel and extensive van der Waals interactions of the C18 alkyl chains with the binding site including residues in helices 4 and 5. Mean docking free energies were 8.73–8.93 kcal/mol for SPH and 8.56–8.15 kcal/mol for DMS, and calculated binding constants were ~40 nM for SPH and ~120 nM for DMS. We hypothesize that SPH, DMS, and similar sphingoid bases access the S1R beta barrel via a membrane bilayer pathway. We further propose that the enzymatic control of ceramide concentrations in intracellular membranes as the primary sources of SPH dictates availability of endogenous SPH and DMS to the S1R and the subsequent control of S1R activity within the same cell and/or in cellular environments.

## 1. Introduction

The Sigma-1 Receptor (S1R) is a unique 223-amino-acid ligand-regulated neuromodulatory/chaperone-like [1] integral membrane protein that responds to cellular stress to preserve homeostasis [2]. Ligands that bind to the S1R regulate conversion of oligomeric (antagonist) to protomeric (agonist) forms [3,4]. This biochemical mechanism is similar to soluble small heat shock protein (sHSP) chaperones in which large oligomer-to-dimer conversions are activated by cellular stress (heat shock) instead of ligands [5,6,7,8]. The S1R is located mainly in specialized intracellular endoplasmic reticulum (ER) membranes (mitochondrial-associated membranes (MAM)) [1], but also in the ER, lysosomal, golgi, nucleoplasmic reticulum [9], and plasma membranes [10]. The S1R is associated with a plethora of eukaryotic signaling pathways involved in peripheral [11] and CNS neuroprotection, neurogenesis [12], neuropathic pain [13,14], cellular oxidative stress [10], obesity [15,16], inflammation, autophagy [17,18,19], and retinal degeneration [20]. These cellular outcomes are underwritten, in part, by direct or indirect molecular associations of the S1R with various membrane-bound “client” proteins such as G-protein-coupled receptors (GPCRs); voltage-dependent sodium, potassium, chloride, and calcium channels; NMDA receptors [21]; dopamine transporters (DAT); transient potential activated (TRP) channels [22]; transcription factors [5,23,24,25]. An impressive number of small molecules have been synthesized that target the S1R [26,27,28,29,30,31,32]. Such drugs, generally identified by S1R binding, functional studies, and in silico and/or structural approaches, are viewed as potential treatments for neurodegenerative human conditions [33,34] such as Amyotrophic Lateral Sclerosis (ALS), retinal degeneration, Alzheimer’s, Parkinson’s Disease, and Huntington Disease. Prevailing interests continue for the identification of endogenous regulators of the S1R that could lead to a better mechanistic understanding of constitutive S1R control(s) [35,36,37,38].

**Figure 1:**
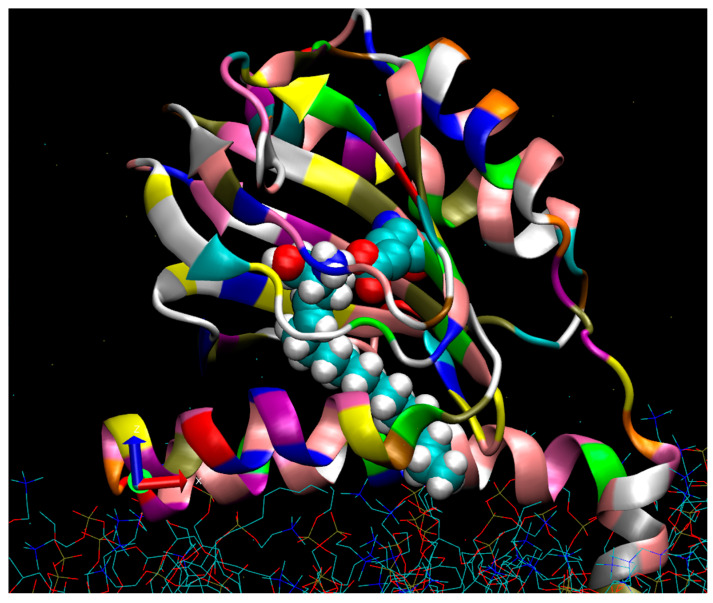
A side view of the human Sigma-1(hS1R) protomer viewed from the lumenal ER bilayer. A molecular modeling extension of a sphingosine (SPH) pose (with an extended C18 alkyl chain) is shown while bound to the hS1R cupin-like beta barrel in a conformation that inserts the alkyl chain between helices 4 and 5. The membrane (blue and red sticks at the figure bottom) was prepared with the program, Charmgui. The S1R is shown with the cupin-like binding site domain located in the ER lumen (center) and the N-terminal helix 1 bilayer imbedded (bottom right). Molecular dynamics (MD) minimization placed the S1R at a slight angle to the membrane surface. As shown, the space-filling SPH tail (with white hydrogens) partially exits to the bilayer between helix 4 (foreground) and helix 5 (behind helix 4). The SPH amino group (blue) is hydrogen-bonded to Glu 172 (turquoise), and oxygen atoms are in red. At least two pathways for the entry of small-molecule regulators into the active site, pathway 1 and pathway 2, have been proposed [40,49]. Pathway 1 features mainly an aqueous entry involving the opening of a “lid” to expose the beta barrel sequestered binding site [50]. Pathway 2 provides for possible bilayer access to the binding site, especially for endogenous sphingoid bases such as proposed for SPH and *N*,*N*’-dimethylsphingosine (DMS) but also for exogenous hydrophobic/amphipathic small molecules.

A homotrimeric crystal structure of the Human [39] and Xenopus [40] S1Rs has been solved utilizing lipidic cubic phase approaches. The S1R is a Type II membrane protein containing an N-terminal signal anchor sequence, a short amino acid sequence facing the cytoplasm, followed by a single N-terminal transmembrane helix (TM1) [41,42]. The remainder of the C-terminus when “spooled” through the translocon into the ER lumen provides S1R-mediated MAM regulation of calcium signaling and interactions in the ER with the intraluminal protein, GRP78/BIP [43], and the IP type3 receptor [1]. A cupin-like beta-barrel fold with an area of approximately 735 cubic angstroms constitutes the S1R active site. Each protomer binding site is supported and stabilized by beta sheets and four helices (2–5) that constitute the bulk of the protein in the ER lumen. The identity of the ligand binding site region from crystal structures has generally confirmed previous biochemical analyses using covalent photoreactive radio-iodinated ligand probes of the native membrane-bound receptor [44,45]. A significant feature of the receptor is the positioning of the two relatively hydrophobic helices, 4 and 5. These two helices appear in an apparently semi-parallel juxtaposition and likely partially imbedded in the membrane (Figure 1). In addition to the protomer form (approx. 25 kDa), the native membrane-bound, epitope and non-epitope-tagged rat, guinea pig, and human-purified S1R receptors occur in multiple oligomeric forms ranging from 50 kDa to >400 kDa. S1R antagonists stabilize the oligomer forms, whereas S1R agonists dissociate the oligomers to smaller forms including the protomer form [3,4,39,44,46,47,48].

**Figure 2:**
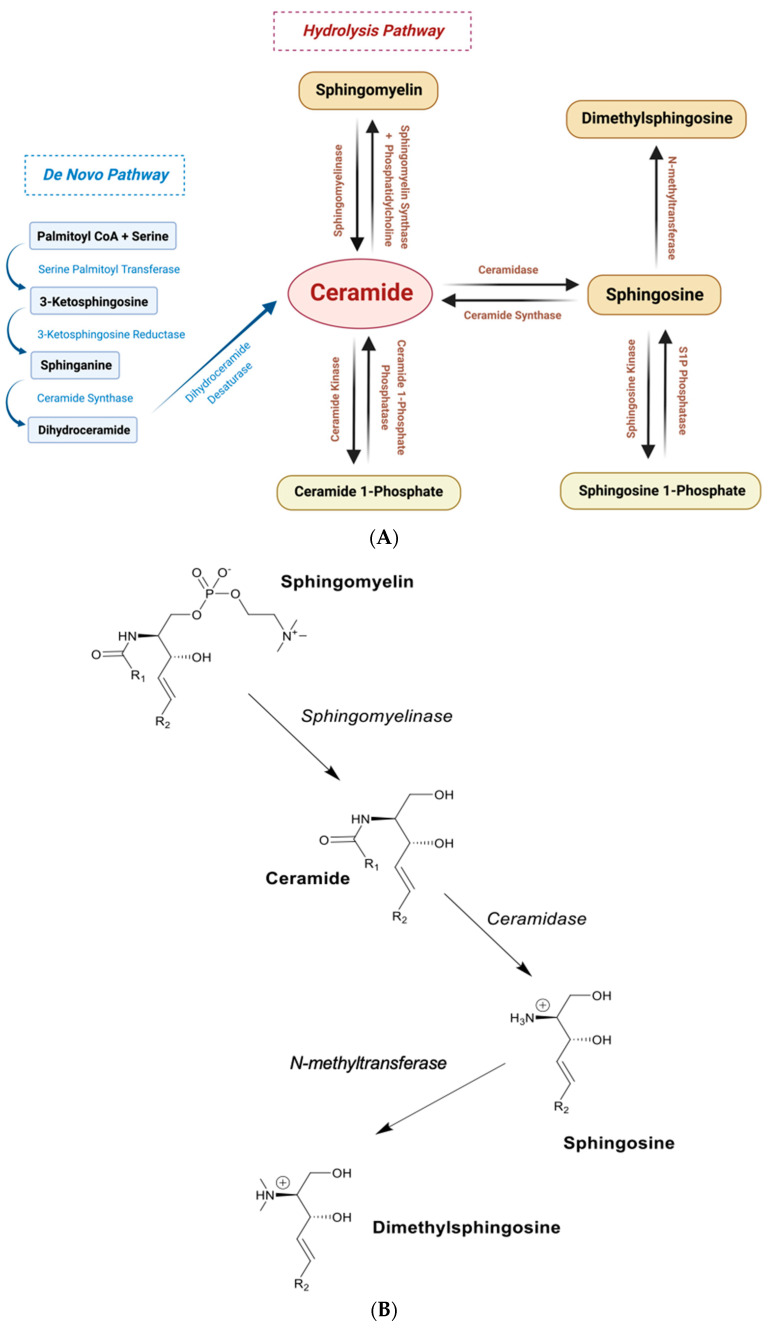
Summary of the metabolic pathways for DMS formation. (**A**) The de novo and the hydrolysis pathway for generation of ceramide. Ceramidase and ceramide synthase activities determine the net formation of SPH from the hydrolysis pathway depending on the intracellular membrane locations and the prevailing conditions. Three forms of alkaline ceramidases (ACERs) occur. ACER1 is localized to the ER as a calcium-activated multi-transmembrane enzyme. ACER 3 is localized to the ER and Golgi complex. (**B**) The Hydrolysis Pathway from sphingomyelin to DMS. Dimethylation to form DMS occurs via an uncharacterized S-adenosylmethyl-L-methionine (SAM) methyltransferase. An intermediate in the formation of SPH and its bioactive derivatives is ceramide. R1 designates C2 to C24 alkyl chains; R2 designates 13 additional carbon atoms to produce C18.

Several exogenous compounds that contain amines and extended N-substituted alkyl chains bind to the S1R [51,52,53,54,55] (Figure S1A) as well as endogenous molecules with similarly extended N-substituted alkyl chains (Figure S1B). The binding kinetics for SPH, DMS, L-threosphingosine, and D-erythrosphinganine are competitive when measured against the agonist, (+)-pentazocine (Kis approx. 100 nM with the pure guinea pig S1R) [55]. Ceramide (Figure 2A,B) and ceramide 1-phosphate failed to inhibit pentazocine binding. SPH co-immunoprecipitated with the S1R from detergent-solubilized HEK293 cell membranes [51]. Specific covalent photoaffinity labeling of the guinea pig liver S1R with (^125^I)-iodoazidcocaine [55,56] was protected by both SPH and DMS but not by ceramide or ceramide 1-phosphate, suggesting that a single sphingoid alkyl chain (as in SPH) but not two alkyl chains or the presence of phosphate negative charges is tolerated in the S1R binding site.

SPH is an important endogenous lipid mediator variously involved in multiple signal transduction pathways [57]. Many bioactive forms of SPH derivatives have been identified, for example, 1-galactosylsphingosine (Psychosine) [58], 1-glucosylsphingosine (glucopsychosine) [58,59], and sphingosine 1-phosphate (S1P) [60]. Signal transduction via S1P occurs through activation of a family of GPCR S1P receptors that generally support the “Sphingolipid Reostat” [61,62,63,64] involving SPH, S1P, specific membrane-bound transporters, and a family of GPCRs. DMS (Figure 2B) formation was originally identified in crude mouse brain homogenates [65], subsequently in cultured inflammation-stressed rat oligodendrocytes [66], and in human multiple-sclerosis-derived oligodendrocytes [67]. A sphingosine N-methy transferase has yet to be purified or otherwise identified. DMS is a bioactive pain-provoking sphingoid [68]. A ceramidase inhibitor, N-oleoylethanolamine, when injected intrathecally into rats after sciatic nerve ligation or administration of DMS, resulted in loss of pain, consistent with a pathway to DMS involving ceramide cleavage by ceramidases [69]. Wei et al. [70] further supported the DMS/pain connections using chronic intrathecal administration of DMS in male adult rats. DMS-induced responses involved TRPM3 ion channels that were blocked by the S1R antagonist, BD-1047. These results may also explain previous observations [71] showing activation of TRPM3 channels by SPH and DMS. Igarashi summarized several features of DMS metabolic control including (either directly or indirectly) through inhibition of protein kinase C [72]. Yatomi et al. [73] further demonstrated inhibition of sphingosine kinase (SK), thus regulating S1P pathways. Additionally, DMS showed activation of the EGF receptor kinase [74] and inhibition of store-operated Ca^2+^ in monocytes [75].

Herein, we demonstrate, via an oligomerization assay, using an intact retinal pigment epithelial (RPE) cell platform and modified native gel analyses that sphingoid bases such as SPH and DMS are endogenous agonists of the S1R. We show, via in silico modeling and molecular dynamics, free energy determinations, calculated affinities, and the molecular characteristics of SPH and DMS interactions with the human S1R (hS1R) protomer binding site. We posit that the pain-provoking sphingolipid, DMS, targets the S1R following *N*,*N*-dimethylation of SPH under pain-provoking conditions in the dorsal root ganglia of rats and in human oligodendrocytes. We hypothesize that the basal and cellular-stress-activated levels of S1R regulation, supported by SPH and DMS, may depend on “local” sphingomyelinase/ceramidase/ceramide synthase enzyme activities that regulate ceramide concentrations in the MAM, ER, golgi, nucleoplasmic reticulum and plasma membranes. Finally, we propose that SPH and DMS access the S1R binding site through “Pathway 2” via the membrane bilayer [40,49].

## 2. Results

### 2.1. SPH and DMS Destabilize S1R Oligomers

**Figure 3:**
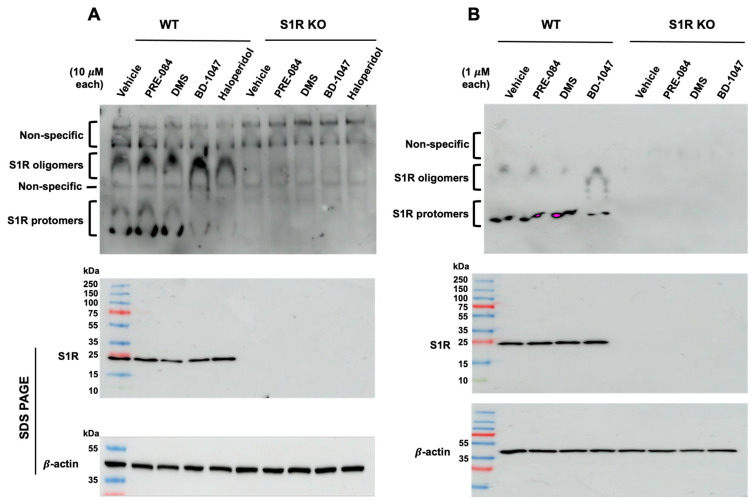
Comparison between WT and S1R KO cells highlights S1R-specific bands on immunoblots. Wild-type and S1R KO human ARPE-19 cells were cultured to full confluency and incubated with vehicle (equal volume of DMSO), BD1047, haloperidol, PRE-084, or DMS at indicated concentrations for 3 h. The cells were lysed in RIPA buffer and mixed with loading buffer. Each sample of 60 μg (**A**) or 25 μg (**B**) was loaded without heating and subjected to non-denaturing PAGE (4–16% polyacrylamide gel) followed by S1R immunoblotting (see the two upper blots). On a separate gel, samples were subjected to standard 10–15% gradient SDS-PAGE and immunoblotting to detect S1R and β-actin. Pre-stained molecular weight markers were used.

The membrane-bound and purified S1Rs exist as a range of molecular sizes from protomers (approx. 25 kDa) to oligomers (approx. 50–400 kDa) [3,39,44,48]. In the presence of S1R antagonists (e.g., haloperidol, BD1047), the oligomeric forms predominate, whereas in the presence of S1R agonists (e.g., pentazocine, PRE-084), smaller sizes predominate [3,4,76]. Recent studies have elegantly demonstrated that S1R agonists and antagonists could be distinguished through non-denaturing PAGE (also known as native gel) followed by S1R immunoblotting [17,76,77,78]. Of note, even a lower-affinity S1R antagonist such as progesterone exhibited a strong oligomer-stabilizing effect [76]. However, the oligomer-destabilizing effect of lower-affinity putative S1R agonists (e.g., DHEA) could not be readily discerned [76]. Moreover, S1R oligomers on blots typically appear as a broad “smeary” band [17,76,77]. It is, thus, impractical to distinguish an S1R-specfic signal from “false-positive” background or S1R homomers from S1R heteromers complexed with other proteins. Herein, we conceived a modified approach. We ran native gels for S1R immunoblotting but for cell lysis, and we used RIPA buffer containing a low amount (0.1% *w/v*) of sodium dodecyl sulfate (SDS) that destabilizes protein complexes. We surmised that inclusion of SDS should reduce the oligomer-stabilizing potency of an antagonist (that is usually high affinity), hence enhancing the relative strength of a lower-affinity agonist to compete against the antagonist. As such, our strategy was to first establish that BD-1047, a high-affinity (Ki = 0.93 nM) and highly S1R-selective antagonist [79], can still maintain S1R oligomers that are weakened by the presence of SDS in the RIPA buffer, and then to determine whether relatively lower-affinity S1R endogenous sphingoid lipids such as SPH and DMS (Ki values for purified S1R of approx. 100 nM) [55] act as agonists, based on whether they can overcome the oligomer-stabilizing effect of BD-1047. We used ARPE-19, a human retinal pigment epithelium (RPE) cell line that expresses the S1R [9,80] as a platform for our experiments, because of the availability of the S1R knockout (KO) ARPE-19 cell line that we generated using the CRISPR/Cas9 technology [9]. As shown on the Figure 3A immunoblot, we were able to detect three well-separated upper, middle, and lower regions that showed positive immunoblotting with antibody against the S1R. Moreover, we were able to “purge” the non-S1R protein upper region from S1R-specific protomers and oligomers. These bands of apparent high molecular weight appeared to be non-specific as they were detected essentially equally in the samples of both WT and S1R KO cells. It is unlikely that they resulted from an antibody quality issue because immunoblotting following SDS-PAGE (bottom) showed a single S1R band and a clean background on the entire blot from the 250 kDa upper region through to the 10 kDa gel front (Figure 3A,B). Consistently, the non-specific background arising from the native gel was diminished if the sample amount was reduced to 25 μg protein per lane (Figure 3B). For enhanced oligomer band intensity, we used a higher loading amount of 60 μg of total cell protein per lane throughout this study. Importantly, the cells treated with the S1R antagonists, BD-1047 or haloperidol (the latter is non-selective for S1R/S2R) [81], manifested an oligomer-stabilized state; that is, more oligomers and less protomers. In stark contrast, vehicle control, DMS, and PRE-084 were associated with an oligomer-destabilized state that showed more protomers and less oligomers. The patterns of immunoblot bands were distinct between cells treated with BD-1047 and PRE-084 at either 10 μM or 1 μM concentrations (Figure 3A,B). While BD-1047 was used as a model S1R antagonist, PRE-084 served as a highly S1R-selective positive-control agonist [26]. The high-affinity and high-selectivity S1R agonists, (+)-pentazocine and/or SKF 10,047 [5,26,81], could not be used in this work, because of University of Virginia institutional regulations regarding controlled substances.

**Figure 4:**
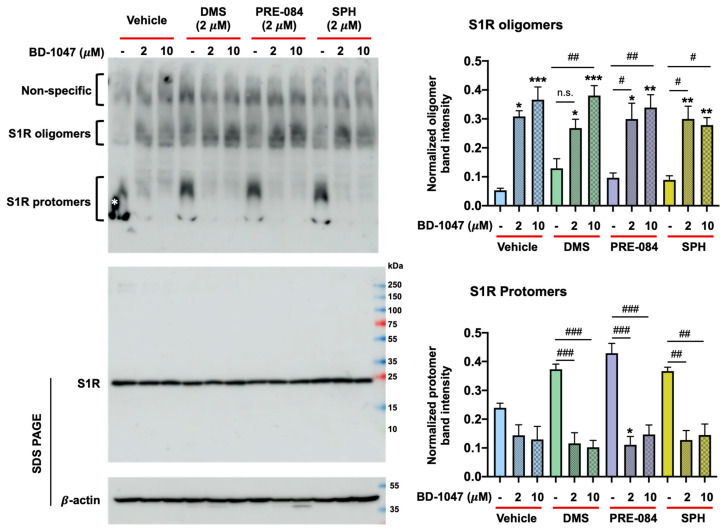
Distinct S1R-agonist- and antagonist-associated patterns of S1R protomer and oligomer bands. Wild-type human ARPE-19 cells were cultured to full confluency and treated with vehicle (equal volume of DMSO), BD-1047, PRE-084, DMS, or SPH with the indicated concentrations, or co-treated with BD-1047 for 3 h. The cells were lysed in the RIPA buffer and mixed with the loading buffer. Each sample of 60 μg was loaded without heating and subjected to non-denaturing PAGE (4–16% polyacrylamide gel) followed by immunoblotting (see the upper blot). On a separate gel, samples were subjected to standard 10–15% gradient SDS-PAGE and immunoblotting to detect S1R and β-actin (lower blots); pre-stained molecular-weight markers were also loaded. The white * in the first lane marks a “bleb” of a non-specific signal possibly caused by a gel running edge effect. Quantification (see the two bar graphs on the right derived from native PAGE experiments): In each lane, the densitometry intensity of oligomers or protomers was measured using Image-J and normalized to that of the total S1R-positive bands in the same lane. The values from repeat experiments were averaged to calculate the mean ± SEM (n = 3 independent repeat experiments). One-way ANOVA and the Bonferroni test were applied. ^#^ *p* < 0.05, ^##^ *p* < 0.01, ^###^ *p* < 0.001 compared to the condition without BD-1047. * *p* < 0.05, ** *p* < 0.01, *** *p* < 0.001 comparing each condition to basal as represented in the first bar (blue) of the plot. n.s.—not significant.

To further establish the oligomer-stabilizing role of the S1R antagonist BD-1047 and to test the putative protomer-enhancing agonist activity of SPH, PRE-084, and DMS, we repeated experiments with BD-1047 at 10 μM or 2 μM. These concentrations of BD-1047 are commonly used in cellular assays to avoid non-specific effects [76,82,83]. As evident in Figure 4, treatment of ARPE-19 cells with either 10 μM or 2 μM BD-1047 markedly increased S1R oligomers compared to vehicle alone. On the other hand, 2 μM SPH, PRE-084, and DMS, when added alone, showed a trend of enhancing protomer formation compared to vehicle alone (*p* > 0.05). BD-1047, when added together with 2 μM SPH, PRE-084, or DMS, blocked (or reversed) protomer formation to form oligomers as expected for agonist/antagonist actions at the S1R. Thus, the quantified data in Figure 4 established that the S1R antagonist BD-1047 could stabilize S1R oligomers (even with 0.1% SDS included in the cell lysis buffer) and that PRE-084, DMS, and SPH produced the same predominant immunoblot protomer patterns consistent with specific agonist-induced S1R activities blocked by a specific antagonist.

**Figure 5:**
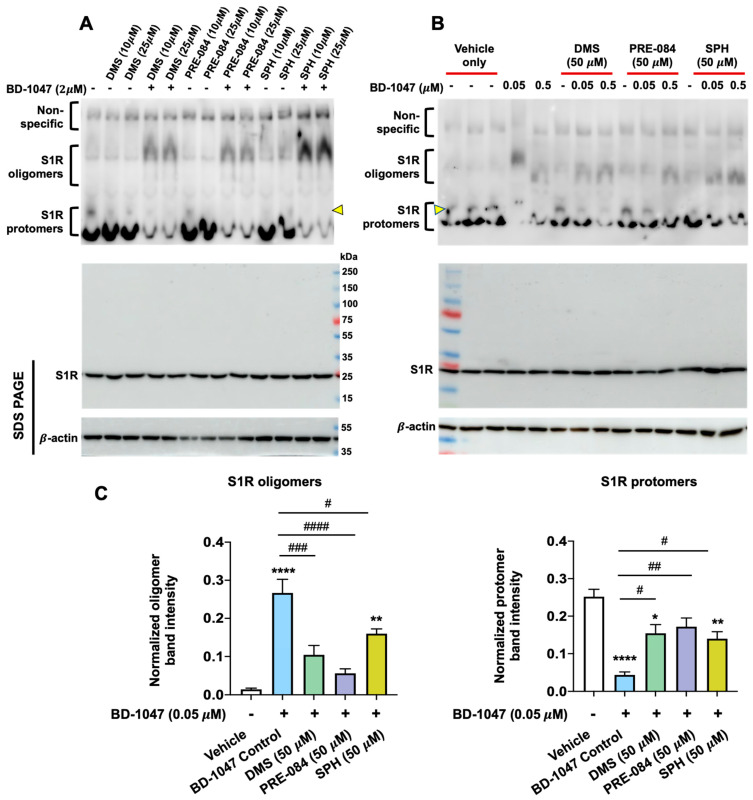
PRE-084, DMS, or SPH in molar excess partially overcomes the oligomer-stabilizing effect of BD-1047. Wild-type human ARPE-19 cells were cultured to full confluency and treated for 3 h with vehicle (DMSO), BD1047, PRE-084, DMS, or SPH alone or with PRE-084, DMS, or SPH each in a 5-fold or 12.5-fold molar excess (**A**) or in a 100-fold or 1000-fold molar excess (**B**) over BD-1047. The cells were lysed in the RIPA buffer and mixed with the loading buffer. Each sample of 60 μg was loaded without heating and immunoblotted following non-denaturing PAGE (4–16% polyacrylamide gel, see upper blots). Standard 10–15% gradient SDS-PAGE and immunoblotting were performed to detect S1R and β-actin (see lower blots). Molecular weights are indicated by pre-stained markers. Yellow triangles mark the position where the bands are consistent with a possible S1R dimer based on Hong’s report [76]. Shown in (**C**) is the quantification performed for the native PAGE experiments (see (**B**), upper right blot) with PRE-084, DMS, or SPH (each 50 μM) in 1000-fold excess over BD-1047 (0.05 μM). In each lane, the densitometry intensity of oligomers or protomers was measured by Image-J and normalized to that of the total S1R-positive bands in the same lane. The values from repeat experiments were averaged to calculate mean ± SEM (n = 4 independent repeat experiments). “BD-1047 control” refers to 0.05 μM BD-1047 alone without adding an agonist. One-way ANOVA and the Bonferroni test were applied. ^#^ *p* < 0.05, ^##^ *p* < 0.01, ^###^ *p* < 0.001, ^####^ *p* < 0.0001 compared to BD-1047 Control (blue bar). * *p* < 0.05, ** *p* < 0.01, **** *p* < 0.0001 comparing each condition to basal as represented in the first bar.

The data reported in Figure 4 represent the usual “forward approach” for assessing small-molecule agonist/antagonist properties of a receptor; that is, agonist activity (S1R protomer formation by SPH, DMS, and PRE-084) and competitive reversal by an antagonist (S1R oligomer formation by BD-1047). Based on the fact that modified native gels produced an excellent and interpretable resolution of oligomers and protomers, we sought to further strengthen the data by using a “reverse approach” strategy. This experiment assesses the “reversal” of BD-1047-stabilized S1R oligomers to protomers by co-incubation with SPH, DMS, and PRE-084 and, thus, importantly, sets the BD-1047 oligomer forms as the experimental “control”. The challenge, as can be readily appreciated, is that BD-1047 with a S1R Ki of less than 1 nM must be overcome, in the steady state, by SPH and DMS that have S1R Kis of approx. 100 nM [55]. This experimental approach required meeting the challenge of testing a series of agonist vs. antagonist stoichiometries with the knowledge of small-molecule accessibilities, especially lipid sphingoid bases and amino-containing compounds that may be trapped in acidic environments (e.g., lysosomes) in intact cells. Accordingly, increasing ratios of SPH or DMS (with PRE-084 as agonist control) were tested vs. BD-1047 at 5:1 (10 μM: 2 μM), 12.5:1 (25 μM: 2 μM; see Figure 5A), and 100:1 (50 μM: 0.5 μM; see Figure 5B). SPH and DMS showed a graded reversal of the “control” BD-1047 oligomer (see the S1R-specific band intensity as divided between protomers and oligomers; Figure 5B). To further increase the ratio to 1000:1, we used 0.05 μM BD-1047 and SPH, DMS, or PRE-084 at 50 μM each, and repeated the experiments for statistical analyses. The quantified data (Figure 5C) indicated that, compared to vehicle only (the first bar), BD-1047 alone (BD-1047 Control) increased S1R oligomers by ~25 fold (**** *p* = 0.00007) and reduced S1R protomers by ~6 fold (**** *p* = 0.00006), indicating its oligomer-stabilizing function. SPH, DMS, and PRE-084 reduced oligomers by ~2–6 fold (^#^ *p* = 0.02, ^###^ *p* = 0.0006, and ^####^ *p* = 0.00008, respectively) and increased protomers by ~3–4 fold compared to the BD-1047 Control (^#^ *p* = 0.03, ^#^ *p* = 0.01, and ^##^ *p* = 0.003, respectively). These results demonstrated that the three S1R ligands (SPH, DMS, and PRE-084) tested could effectively “de-stabilize” the BD-1047 S1R oligomers, thereby exhibiting “reverse”-agonist-mediated responses. Taken together, the findings indicate that, compared to PRE-084, SPH and DMS fulfill the requirements as endogenous S1R agonists in the enhanced native gel assays.

### 2.2. SPH and DMS Dock to the S1R Cupin Beta Barrel

To further support the results obtained through the native gel approach, we performed molecular docking. The two endogenous sphingoid compounds, SPH and DMS, were docked as the controls in the 6DJZ receptor. In all dockings, the protein was static and the sphingoid lipid was randomly torsioned and then docked into the protein in the defined box. As seen in Tables S1 and S2, the docking energies for the two compounds did not show a large difference in energies in the largest clusters for each sphingoid base. An initial docking was performed for SPH and DMS, and the two best dockings were re-docked to confirm that the best dockings were not from chance. For the first docking of SPH, the largest cluster of 23 out of 30 had a mean binding energy of −8.73 kcal/mol with the best fit binding energy of −10.15 kcal/mol. For the first docking of DMS, for the largest cluster of 15 out of 30, the mean energy was −8.56 kcal/mol with a best fit of 9.43 kcal/mol. For the re-docking, the results were SPH: 22/30, mean: −8.93, and best fit: −10.11 kcal/mol. For re-docking, DMS: 21/30, mean: −8.15, and best fit: −9.43 kcal/mol. Notably, the torsional free energy of both molecules was +5.37 kcal/mol and was subtracted from the total negative energy from total intermolecular energy and internal energy. For example, for SPH in the first docking, the sum was (−13.91) + (−2.36) + (+5.37) − (0.75). The total intermolecular energy was approximately −14 kcal/mol. The estimated Kis, as determined from the best free energies, were 36.6 nM and 38.68 nM for SPH (Table S1) and 122.4 nM and 122.88 nM (Table S2) for DMS. The calculated binding constants obtained from the docking free energies were, therefore, roughly in the same range (around 40–100 nM) as those measured by competitive displacement assays on the purified guinea pig S1R using tritiated (+)-pentazocine [57].

### 2.3. Molecular Dynamics of SPH Docking to the S1R

The 200-nanosecond MD was initiated with the best docking fit that has a value of −10.15 kcal/mol (Tables S1A and S2B). Beginning with H bonding to the Asp126 (space-filled gold-colored), the charged nitrogen maintains contact with the Asp126 throughout most of the 200 nanoseconds and is, thus, in a stable position between His154 (space-filled blue and red) and Asp126. The calculated volume of a Connelly surface (water-accessible) of SPH is 282 cubic angstroms and occupies 282/732 or 38.5% or roughly 1/3 of the pocket volume.

### 2.4. Molecular Dynamics of DMS Docking to the S1R

A 200-nanosecond run of DMS was initiated with one of the top positions in the docking modes (Tables S2A and S2B) The dimethylamino group position begins with hydrogen bonded to Asp126 (space-filled gold). The methyl groups remain in a stable orientation, precluding His154 (space-filled blue and red colored) from interacting. The charged nitrogen group switches to the carboxylate of Glu 172 (space-filled silver) at approximately 50 nanoseconds and remains attached until the 200 nanoseconds run is completed. There is a brief time that the DMS nitrogen disassociates from Glu172, but it quickly reassociates and remains stable in that pose for the remainder of the 200 nanoseconds.

## 3. Discussion

### 3.1. SPH and DMS Are S1R Agonists

While evidence from our laboratories and others’ implicated SPH and DMS as S1R agonists [55,76], this was never clearly defined. Herein, we were able to identify two naturally occurring sphingoids as S1R agonists by using a modified native gel approach. To detect S1R oligomer-destabilizing effects of lower-affinity S1R ligands, we conceived an approach of abating the strong oligomer-stabilizing potency of BD-1047 by including a low amount of SDS in the cell lysis buffer. We were, thus, able to use the endogenous lipids, SPH and DMS, in excess, to overcome BD-1047 and read out their oligomer-destabilizing agonist effects. This strategy differs from the traditional receptor approaches of using antagonists to block agonist functions. While our experiments were sufficiently sensitive to detect the agonist activity of SPH and DMS that are lower-affinity ligands (Kis, approximately 100 nM) for the purified guinea pig liver S1R [55], we were also able to resolve S1R protomers and oligomers into relatively “non-smeary” bands that were well separated from the non-specific background. In addition, a higher band within the protomer region, e.g., marked by a yellow triangle on Figure 5 blots, could be discerned, which is consistent with a possible S1R dimer based on Hong’s report [76], although more research is required to verify this possibility. As seen here and in previous studies [76], it is technically challenging to determine the oligomer-destabilizing effect of lower-affinity putative agonists. There are at least two possible reasons for this. First, BD-1047 has a very high affinity for the S1R (Ki < 1 nM) [79], thus requiring much higher concentrations of naturally occurring S1R agonists to overcome the antagonist effect. We estimate that the affinities of naturally existing molecules such as SPH, DMS, and DHEA are at least 100- to 500-fold lower [26,55] than that of BD-1047, although this comparison could be different in assays using intact cells, considering the influence of intracellular signaling pathways on antagonist-S1R interactions [84]. Along this line of thought, PRE-084 has a very high affinity (Ki = 2.2 nM) to S1R in isolated membranes [85], but it is slow and inefficient in reaching the S1R binding site when incubated with intact cells [77], thus possibly reducing its efficacy. Second, endogenous S1R agonists other than SPH and DMS have been reported [26,35,38,81] that may pre-occupy the S1R ligand binding site. Technical aspects of native gel oligomer/protomer assays, regardless of measured binding affinities of S1R ligands, are compounded by detergents (e.g., perfluorooctanoic acid [76,77,78], sodium lauroyl sarcosinate [76], and SDS) often used for sample preparation and/or PAGE. Therefore, as both agonists and detergents can destabilize S1R oligomers, the investigator is challenged in the assessment of further stabilization by an added agonist. This may account at times for the lack of a significant difference between the vehicle control and agonist alone in S1R oligomer/protomer native gel assays. Thus, in order to sensitively read out the S1R oligomer-destabilizing agonist function, we used the antagonist BD-1047 as the control (Figure 5) to stabilize S1R oligomers to a level significantly beyond basal control levels, and then “re-destabilized” the oligomers with candidate endogenous agonists such as SPH and DMS.

In conclusion, the simple modified native gel-based method we present herein proves useful for identifying potential S1R agonists, especially those with low affinities for the S1R. We speculate that low concentrations (0.1% *w/v*) rather than high concentrations of SDS [86] can be effectively utilized in native gel S1R oligomer/protomer assays without strongly destabilizing S1R oligomers and without compromising ligand-binding ability. This approach may offer an avenue to study oligomeric S1R inhibitory client protein interactions that are functionally activated by de-inhibition using an S1R agonist [1].

### 3.2. Docking of SPH and DMS to the S1R Cupin Beta Barrel

**Figure 6:**
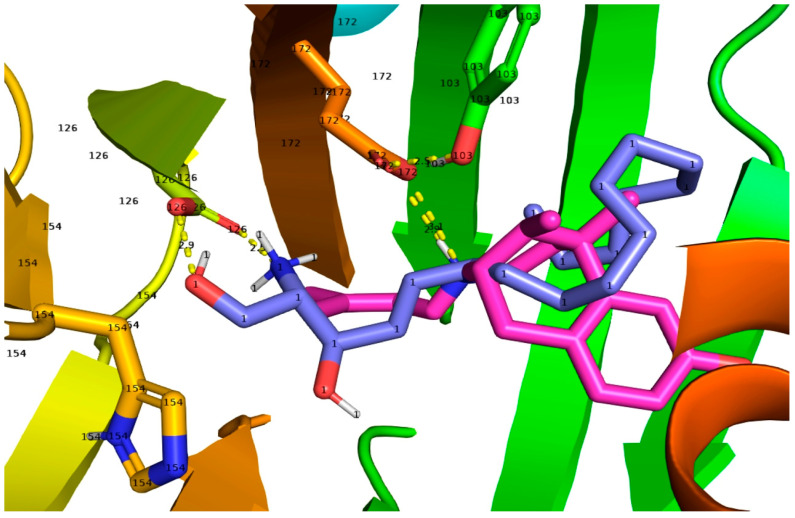
Docking pose of SPH in the crystal structure of the hS1R with (+)-pentazocine also bound (6DJZ). Pentazocine (magenta) in the crystal structure displays a positively charged nitrogen H bonded to Glu172. In the most likely docked structure of SPH (blue), when Asp126 is deprotonated, the protonated nitrogen and the 1-hydroxyl of SPH seek deprotonated Asp126. The hydrophobic tail of SPH is tucked into a mostly hydrophobic region in the protein near helices 4 and 5 that contact the membrane. Clustering and energies for SPH docking can be found in Tables S1 and S2.

**Figure 7:**
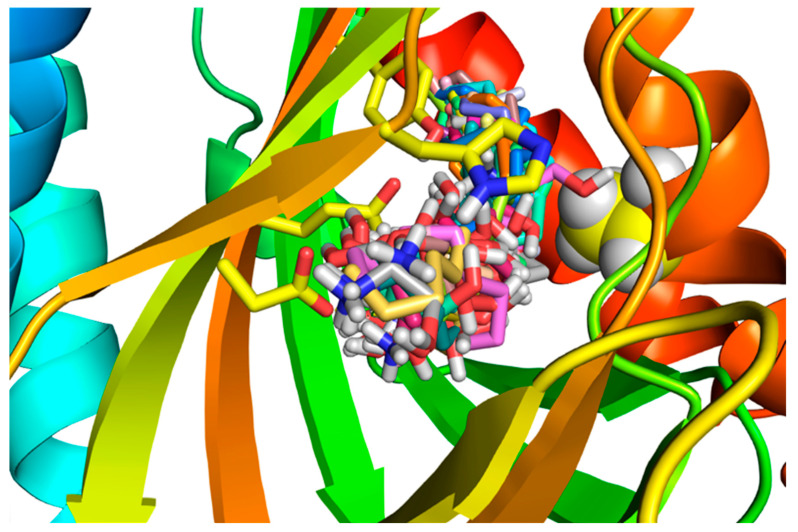
A view of all 30 SPH molecules docked to the human S1R beta barrel. The Pymol figure shows the rainbow blue N-terminal to the red C-terminal Helix 5. The relevant side-chains that interact are in yellow sticks: Asp126, Glu172, Tyr103, and His154. Ala185 on the orange Helix 4 that appears to interact with (+)-pentazocine is colored in space-filling yellow with silver hydrogens, showing the close interaction of Ala185 with the SPH poses and possibly altering the structure of Helix 4. Hydrophobic interactions in the binding site with the alkyl chains of both SPH and DMS (Lig plot Figs in Tables S1 and S2) and in MD simulations showed interactions with S1R side-chains of the alpha 4/alpha 5 helices that likely enhance their agonist efficacies (Tables S1 and S2, and MD). The influence of long alkyl chains in the promotion of S1R activation efficacies is consistent with agonist properties attributed to the endogenous carboxylic acid, myristic acid (tetradecanoic acid) [87], dialkylamines [52,53], and *N*-[3-(4-nitrophenylpropyl) alkyl-1 amines [54]. On the other hand, endogenous S1R agonists that lack alkyl chain S1R binding site interactions as shown for SPH and DMS (see Lig Plots in Tables S1 and S2) such as *N*,*N*’-dimethyltryptamine (DMT) [35], choline [38], and neurosteroids [37,88,89] generally show lower binding affinities and require higher concentrations for functional efficacies.

The characterization of SPH and DMS as an S1R agonist via a modified native gel approach is consistent with our molecular docking results. The best fit poses for SPH and DMS compared favorably to that of the agonist, (+)-pentazocine, as shown for comparison with SPH (Figure 6), in the human S1R crystal structure [50] and as reported for the agonist PRE-084 in the Xenopus S1R [40]. MD poses for SPH and DMS were followed over 200 nanoseconds and are described in detail in the Methods section. The key residues, Asp172 and Glu126 (and other strategic residues such as His154 and Tyr103) contribute to the binding interactions. Notably, poses of the long alkyl chains of both SPH and DMS extend within 3.4 angstroms of several hydrophobic side-chains (residues 178–185) near the N-terminus of helix 4 and residues (202–206) near the N-terminus of helix 5 (see LIG plots in Tables S1 and S2, and Figure 7), showing a compilation of all 30 poses for SPH in the human S1R binding site. Ala185 appears to partially obstruct the alkyl chains of SPH and may, thus, contribute to a “movement” of helix 4 that could underly a mechanism for the agonist activities of SPH, DMS, and other sphingoid derivatives. Helix 4 as has been previously suggested to be involved in S1R activation by (+)-pentazocine and PRE-084 in the Human [50] and Xenopus S1Rs [40], respectively.

### 3.3. Sphingolipid Microdomains Are Potential Primary Sources of Endogenous SPH and DMS

The roles that ceramides play in membrane sphingolipid biochemistry are extensive and relevant to the manner by which S1R can access sphingoid bases. It is generally accepted, for example, that ceramidases play significant roles in regulating ceramide and sphingosine concentrations in cellular membranes [90,91]. Ceramides contribute mainly, but not exclusively, to the bilayers of MAM, ER, and Golgi [92]; for example, even in late phagosomes, glucosylceramides formed by glucosylceramide synthase (GCase) and UDP glucose are elevated for ceramide processing [93]. A generic signature binding sequence, VAMTLGQIYY, for sphingomyelin [94,95,96] (structure in Figure 2B), has been identified within the TM helix of the Golgi coat protein, COPI p24 [97]. Sphingomyelin/p24 interactions regulate a functionally key monomer to dimer equilibrium. Interestingly, a sequence similar to the sphingomyelin signature occurs in the S1R N-terminus TM 1 helix immediately at the C-terminal of the cholesterol CARC sequence [98]. The sequence is VAAVLTQVVW in the human S1R with equivalents in other species. As ceramides can be formed from sphingomyelin by sphingomyelinase (Figure 2B), this signature may offer pathways for homo-oligomerization and/or client protein hetero-oligomerizations of the S1R and for sphingosine formation from ceramide via possible ceramidase/S1R heterodimerization complexes. Another sequence, which is related to the sphingomyelin signature, occurs in the human S1R within amino acid residues 190–199 (VFSTQDFLTF) in the C-terminus of helix 4 extending into the N-terminus of helix 5. This region of the receptor has been identified as a S1R trimerization interface [39] from the hS1R crystal structure.

In the MAM, ER, and Golgi, the ceramide/cholesterol/PC-related lipid microdomain mixtures, thus, provide a favorable environment to stabilize S1R oligomers and provide access to ceramide synthases and ceramidases [92]. Insightfully, a S1R/cholesterol microdomain has been proposed at the MAM/ER interface via a CARC [99] signature sequence in TM 1 that may create a lipid/protein “interactome” [12,98]. It should be possible to take advantage of the emergence of ceramide synthases and ceramidases as drug targets [63,100,101,102] in order to better understand the role(s) of ceramides, SPH, and sphingoid bases as S1R regulators in cellular homeostasis [90] and in ceramide-related diseased states. The steady-state levels of sphingosine and access to the S1R, therefore, will depend on local ceramide concentrations controlled by dihydroceramide desaturases, sphingomyelinases, ceramide synthases, ceramidases, flipases, methylases, kinases, and phosphatases in the MAM, ER, Golgi-derived, nucleoplasmic reticulum and the plasma membrane. Taken together, it is reasonable to consider that the presence of sphingolipid microdomains in intracellular membrane networks may serve not only as “platforms” [103,104,105] for S1R physical associations, but also as sources of endogenous sphingosine and sphingoid bases as S1R agonists.

### 3.4. SPH and DMS Access to the S1R Bindng Site—A Hypothesis

The S1R binding pocket appears occluded, leading to the conclusion that a “lid” movement is required for ligand entry. However, at least two pathways for ligand entry into the S1R beta barrel have been recently considered [40,49]—Pathway 1 and Pathway 2. Pathway 1, as originally described by Schmidt et al. [50] and further supported by Rossino et al. by using steered dynamics [49], supports the opening of the “lid” and an initial dismantling of the side-chain interactions in the beta barrel including Q135, H154, and E158.

Compelling arguments for hydrophobic amphipathic small-molecule entry into the binding site via the bilayer supporting Pathway 2 [40] have also been proposed. Pathway 2 entry is supported by co-crystal structures of the Xenopus S1R with the S1R agonist, PRE084, as either “trapped” or in transit, between the putative bilayer juxtaposed helices 4 and 5 [40] (Figure 1). The presence of an unknown small-molecule-like density of interest in the “endo-closed” structure of the Xenopus S1R that was expressed and purified from yeast [40] may be a sphingoid base. Pathway 2 is an attractive entry mechanism for endogenous sphingoid S1R agonists such as SPH and DMS as they can be formed locally from ceramide “reservoirs” in the bilayer. A provocative “lipid extraction encapsulation” concept for sphingosine removal from the bilayer for access to the catalytic site of sphingosine kinase SK1 has been advanced [60]. This proposed mechanism involves the opening and closing of two membrane-interacting alpha helices in SK1 (alpha7/alpha 8) that support hydrogen bonding of the sphingosine head groups with a local aspartate and other side-chain “acceptors” in SK1. A similar mechanism may be in play in the region of the membrane juxtaposed alpha 4/5 helices in the S1R that allow for “extraction encapsulation” of sphingosine and/or sphingoid base derivatives and/or exogenous amphipathic compounds. Although sphingosine is a consummate water-soluble amphipath that can transverse bilayers, it is unlikely to be energetically favorable for the hydrophobic long-alkyl-chain sphingoid-like molecules to enter the binding site through Pathway 1. This conclusion is further supported by the relatively polar environment contributed by several residues including His154, Glu126, and Asp172 in the beta barrel, the favorable docking free energies for both SPH and DMS (Tables S1 and S2), and the Kis obtained from binding experiments [55].

In summary, several observations support single-alkyl-chain sphingoid-like bases as endogenous agonists of the S1R: (1) Both SPH and DMS show S1R agonist properties in intact RPE cells (Figure 3 and Figure 4) when interrogated for reversal of S1R oligomeric forms to smaller forms (Figure 5). (2) SPH and DMS dock directly to the S1R beta barrel with free energies and calculated binding constants that are consistent with previously reported (+)-pentazocine competitive binding data. (3) In silico-docked poses for SPH and DMS, and molecular dynamics analyses are consistent with Asp 126 and Glu172 interactions and extensive binding site interactions of the SPH and DMS alkyl chains (Tables S1 and S2, Ligplots). (4) The alkyl chains of SPH, DMS, and other sphingoid bases may alter the orientation of helix 4/helix 5 and contribute to destabilization of oligomer interfaces to yield smaller active forms (Figure 1 and Figure 7). (5) SPH and DMS may access the S1R from the bilayer via Pathway 2 (Figure 1). (6) The ubiquitous (but likely variable) accessibilities of ceramides and ceramidases to the S1R in intracellular and plasma membranes may regulate the “local” availability of SPH and/or sphingoid bases to the S1R.

## 4. Materials and Methods

### 4.1. Wild-Type (WT) and S1R Knockout (KO) ARPE-19 Cell Lines

The human RPE cell line (ARPE-19) was purchased from American Type Culture Collection (CRL-2302, ATCC, Manassas, VA, USA). The generation of the S1R KO ARPE-19 cell line using a CRISPR/Cas9 genome-editing approach was described in our previous report [9]. In brief, the *SIGMAR1*-targeting single-guide RNA (sgRNA) sequence was cloned into lentiCRISPR v2. Packaged lentivirus was used to transduce ARPE-19 cells for 3 days. The cells were selected with 1 μg/mL of puromycin for one week, and serial dilution of the cells was performed for the selection of S1R KO single clones. The cells transduced with the empty vector served as the WT control.

### 4.2. Cell Culture and Treatment with S1R Ligands

For cell culture, we used the DMEM/F12 medium (11320082; Thermo Fisher Scientific, Waltham, MA, USA) supplemented with 10% FBS and penicillin/streptomycin (5140163; Thermo Fisher Scientific). ARPE-19 cells were seeded in six-well plates at 3 × 10^5^ cells/well and cultured to ~90% confluency at 37 °C in a humidified atmosphere with 5% CO_2_. S1R ligands or vehicle control (equal amount of DMSO) were added at indicated concentrations and incubated for 3 h before cell harvest for native gel assays.

### 4.3. Non-Denaturing (Native) Poly Acrylamide Gel Electrophoresis (PAGE)

At the end of incubation with S1R ligands, cells were harvested and lysed in the RIPA buffer (89901; Thermo Fisher Scientific) that contained 25 mM Tris-HCl pH 7.6, 150 mM NaCl, 1% NP-40, 1% sodium deoxycholate, and 0.1% SDS, and were supplemented with Halt Protease Inhibitor Cocktail (87785; Thermo Fisher Scientific). Cell lysates were centrifuged at 20,000× *g* and 4 °C for 20 min. Concentrations of total proteins in cell lysates were measured using the DC Protein Assay Kit (5000111; Bio-Rad, Hercules, CA, USA). Each sample of 60 μg of proteins was mixed with 6 gel loading buffer containing 375 mM of Tris-HCL, pH 8.0, 50% glycerol, and 0.03% bromophenol blue, and loaded into a native gradient (4–16%) polyacrylamide gel without heating. The gel was prepared with 0.5× TBE buffer containing 44.5 mM Tris, pH 8.0–8.5, 44.5 mM boric acid, and 1.0 mM EDTA (RNAase- and DNAase-free). For the 4% gel, we used 0.8 mL of 5× TBE buffer, 1 mL of 30% acrylamide/bis-acrylamide solution (29:1, Bio-Rad), 0.08 mL of 10% ammonium persulphate solution (Sigma-Aldrich, St. Louis, MO, USA), 0.008 mL of TEMED (Bio-Rad), and 6.1 mL of ddH_2_O. For the 16% gel, we used the same recipe except for 4.5 mL of 30% acrylamide/bis-acrylamide solution and 2.6 mL of ddH_2_O. Native PAGE was run in pre-chilled 0.5× TBE buffer at 100 V for 4–5 h in a cold room (4 °C).

For SDS-PAGE, the cell lysates solubilized in RIPA buffer were mixed with a 6-gel loading buffer (375 mM Tris-HCL, pH 8.0, 50% glycerol, and 0.03% bromophenol blue) supplemented with 1 mM DTT and 5% (*v/v*) β-mercaptoethanol and heated at 95 °C for 30 min before loading to a 10–15% gradient polyacrylamide gel.

### 4.4. Immunoblotting

After native PAGE, proteins were transferred onto a PVDF membrane at 300 mA for 2 h in a pre-chilled 1× transfer buffer containing 24.8 mM Tris base, 193.2 mM glycine, and 20% (*v/v*) methanol in a bucket kept on ice. The membrane was first incubated with a S1R antibody (sc-137075, Santa Cruz, Dallas, TX, USA, 1:1000 dilution) overnight at 4 °C, washed 3× with PBS, incubated for 1 h at room temperature with goat anti-mouse IgG (H + L)-HRP conjugate (cat. 1706516; Bio-Rad), washed 3×, and illuminated with Clarity ECL Western blotting substrates (cat. 1705060; Bio-Rad). Immunoblot images were recorded using Amersham Imager 680 (GE Healthcare, Chicago, IL, USA). Densitometry intensities of S1R-positive bands were quantified with Image-J software (Version 1.51 23). Considering that the S1R protein appears to react to its antibody differently on a native-PAGE blot than on an SDS-PAGE blot, we normalized the intensity of S1R oligomers (or protomers) with the intensity of the total S1R-positive signal in the same lane, rather than with the beta-actin band intensity on the SDS-PAGE blot.

### 4.5. Statistical Analysis

Independent repeat experiments of native PAGE followed by immunoblotting were performed 3 or 4 times as specified in figure legends where applicable. Data are presented as mean ± SEM unless otherwise specified. The GraphPad Prism 7 software was used for statistical analysis. A normal distribution, while expected, has not been proven.

### 4.6. SPH and DMS Dock to the S1R Protomer Binding Site

The S1R model protein was taken from PDB entry 6DJZ, S1R with haloperidol. The protein is a trimeric membrane protein containing a cupin fold [50] with each protomer bound to haloperidol and an N-terminal alpha helix that is inserted into the membrane. First, the protein was prepared for docking using the protein preparation tools of Sybyl (Tripos Corp). Missing side-chains were added in structurally reasonable positions. None of these were in the active site. The haloperidol was removed and hydrogens were added. The two carboxylates in the active site, Aps126 and Glu172, were in the deprotonated state. Minor side-chain clashes were repaired visually. Gatseiger–Huckel charges were added and the structure energy-minimized in steps: hydrogens alone, then side-chains and backbone, followed by the whole molecule. The Tripos force field was used with Simplex initial optimization, followed by Powell and terminated when the gradient reached 0.05 Kcal/mole. The molecule was then removed and used for docking experiments.

Dockings were performed with Autodock4 [106] utilizing AutodockTools. A box of approximate dimensions 30 × 30 × 30 angstroms was centered in the cupin pocket and included the majority of the pocket as well as the alpha 4 and alpha 5 helices that are juxtaposed on the membrane surface. This is including His154, Asp126, and Glu172. Both (+) pentazocine, an agonist, and haloperidol, an antagonist, were used as controls to validate that the docking procedure was correct. Haloperidol, with a proton on the nitrogen and, thus, positively charged, was found to dock in a similar way as found in the crystal structure 6DJZ with the nitrogen hydrogen-bonded to Glu172. Autodock produced the top 30 lowest energies and clustered them into groups. The docking reproduced the crystal structure in the top 10 best (lowest energy) examined. Initially, (+)-pentazocine would not dock into 6DJZ, because it interfered with Ala185 in helix 4. When helix 4 was moved slightly and energy-minimized, (+)-pentazocine docked as found in the crystal structure 6DK1, with the top 10 best dockings reproducing the interactions of the positively charged ring nitrogen interacting with Glu172. In both control compounds, Asp126 was deprotonated. The Autodock scoring is an addition of 4 intermolecular-contributing Kcal/mol energy terms as the sum of Intermolecular Energy (VdW + Hbond + desolvation + electrostatics), plus total internal energy, plus torsional energy, minus the unbound system energy. The comparison docking of (+)-pentazocine and SPH is shown in Figure 6. The positively charged nitrogen hydrogen bonded to the Glu172 as expected. In some of the less energetic dockings, the Tyr120 hydroxyl proton is involved in interactions, but Tyr103 hydroxyl is donated to the carbonyl in the interior of the binding site, stabilizing the orientation of the carboxylate on Glu172. The docking of SPH and DMS was performed under the same. The two endogenous sphingolipids, SPH and DMS, docked similarly to the control (+)-pentazocine in 6DJZ S1R. In all dockings, the protein was static and the ligand was randomly torsioned and then docked into the protein in the defined box.

### 4.7. Molecular Dynamics (MD) Performed on Docked SPH and DMS in the Best Energy Position

GROMACS 2020.4 Manual (Zenodo. https://doi.org/10.5281/zenodo.4054996 (accessed on 6 October 2020) was used to run the dynamics and the force field used was Charmm 2020 [107]. cGENff [108] was used to calculate the topology of the lipids. The N-terminal helix was truncated at 28 amino acids, as these N-terminal amino acids were not considered to contribute directly to the energies in the cupin beta barrel. The protein–lipid complexes were fully solvated. The complexes were converted to Gromacs parameters and energy-minimized. Volume and pressure equilibration was performed to stabilize the system before dynamics. The dynamics were performed with electrostatic particle-mesh Ewald summation over the long range. There were 2-femtosecond steps used until the run reached 200 nanoseconds. The dynamics were analyzed and converted to a movie using VMD [109].

## Data Availability

Upon request, molecular dynamics of SPH and DMS docking to the S1R will be available in DVD from contributing authors.

## Supplementary Materials

The following supporting information can be downloaded at: https://www.mdpi.com/article/10.3390/ijms24043103/s1. References [51,52,53,54,55,110,111] are cited in the Supplementary Materials.
