# Supplementary material for: Sphingoid Bases Regulate the Sigma-1 Receptor—Sphingosine and N,N’-Dimethylsphingosine Are Endogenous Agonists
Source: Int J Mol Sci. 2023 Feb 4;24(4):3103. doi: 10.3390/ijms24043103 (PMC9962145; doi:10.3390/ijms24043103)
Supplement: Supplementary file 1 [file ijms-24-03103-s001.zip › ijms-2165715-supplemental materials updated.pdf]

SUPPLEMENTAL TABLES AND ASSOCIATED DOCKED STRUCTURES

Table S1A.

Sphingosine (SPH) 6DJZ S1R (first docking run).

Clus -ter Rank	Lowest Binding Energy	Run	Mean Binding Energy	Num in Clus	Histogram (5 10 15 20 25 30 35)
1	-10.15	14	-8.73	23	#######################
2	-9.29	5	-9.29	1	#
3	-8.70	13	-8.16	3	###
4	-8.54	17	-8.51	2	##
5	-8.44	24	-8.44	1	#

```
MODEL        16
MODEL        14
USER    Run = 14
USER    Cluster Rank = 1
USER    Number of conformations in this cluster = 23
USER
USER    RMSD from reference structure       = 1.900 A
USER
USER    Estimated Free Energy of Binding    =  -10.15 kcal/mol  [=(1)+(2)+(3)-(4)]
USER    Estimated Inhibition Constant, Ki   =   36.60 nM (nanomolar)   [Temperature = 298.15 K]
USER
USER    (1) Final Intermolecular Energy     =  -13.91 kcal/mol
USER        vdW + Hbond + desolv Energy     =  -10.93 kcal/mol
USER        Electrostatic Energy            =   -2.98 kcal/mol
USER    (2) Final Total Internal Energy     =   -2.36 kcal/mol
USER    (3) Torsional Free Energy           =   +5.37 kcal/mol
USER    (4) Unbound System's Energy         =   -0.75 kcal/mol
USER
USER
USER
USER    DPF = SPN1_molA_6djz_recep_min.dpf
USER    NEWDPF move    SPH1.pdbqt
USER    NEWDPF about   11.049500 38.264500 -36.382800
USER    NEWDPF tran0   10.282394 36.294722 -37.057911
```

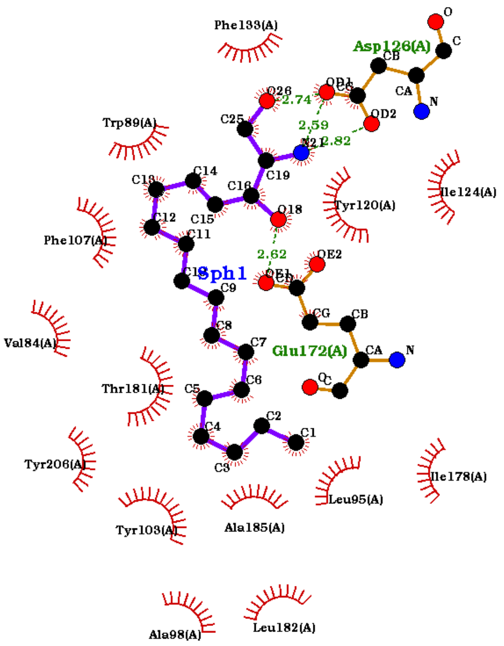

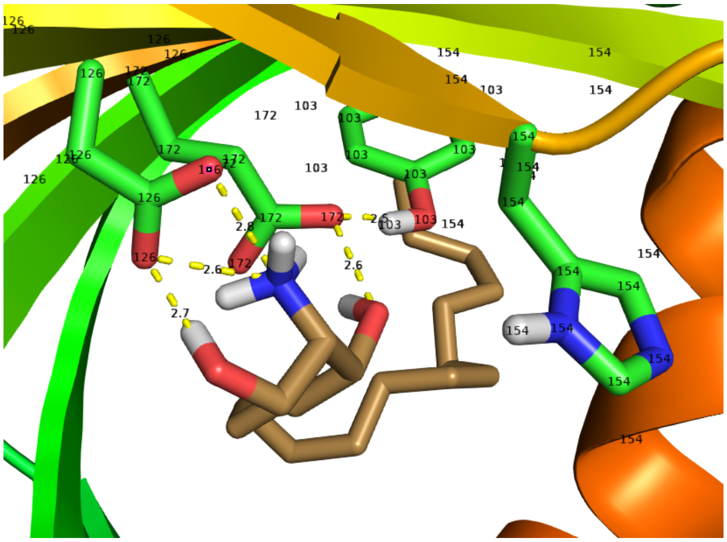

molA-6djz-recep-min_SPH1_1

Table S1B.

Sphingosine (SPH) 6DJZ S1R (second docking run).

Clus -ter Rank	Lowest Binding Energy	Run	Mean Binding Energy	Num in Clus	Histogram (5 10 15 20 25 30 35)
1	-10.11	22	-8.93	22	######################
2	-9.20	30	-8.41	3	###
3	-9.11	13	-8.31	3	###
4	-8.77	2	-8.74	2	##

```
MODEL        22
USER    Run = 22
USER    Cluster Rank = 1
USER    Number of conformations in this cluster = 22
USER
USER    RMSD from reference structure       = 1.498 A
USER
USER    Estimated Free Energy of Binding    =  -10.11 kcal/mol  [=(1)+(2)+(3)-(4)]
USER    Estimated Inhibition Constant, Ki   =   38.68 nM (nanomolar)   [Temperature = 298.15 K]
USER
USER    (1) Final Intermolecular Energy     =  -13.28 kcal/mol
USER        vdW + Hbond + desolv Energy     =  -10.58 kcal/mol
USER        Electrostatic Energy            =   -2.70 kcal/mol
USER    (2) Final Total Internal Energy     =   -2.97 kcal/mol
USER    (3) Torsional Free Energy           =   +5.37 kcal/mol
USER    (4) Unbound System's Energy         =   -0.76 kcal/mol
USER
USER
USER
USER    DPF = SPN21_molA_6djz_recep_min.dpf
USER    NEWDPF move    SPH21.pdbqt
USER    NEWDPF about   11.871500 37.560000 -35.771600
```

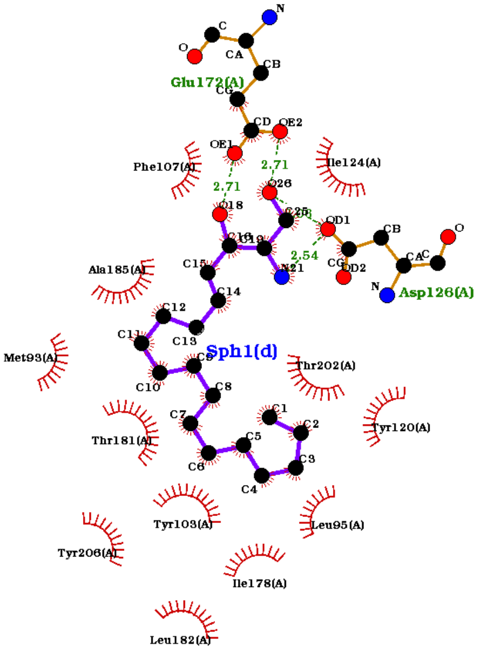

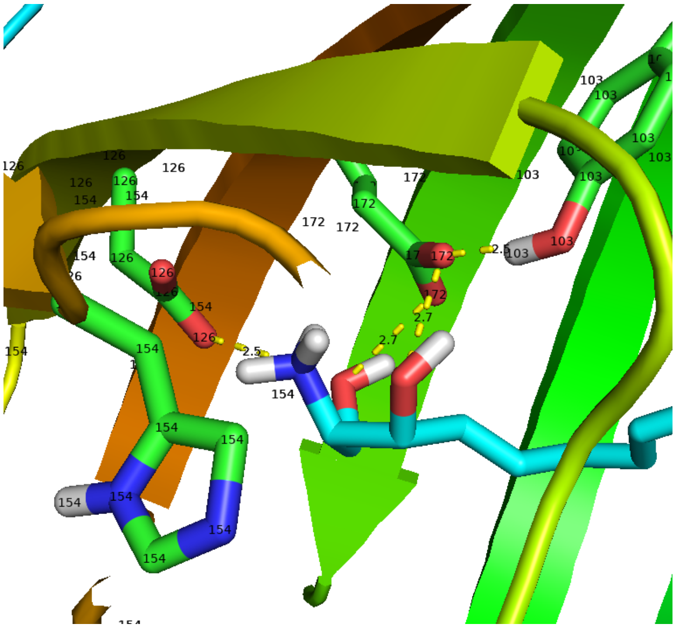

molA-6djz-recep-min_SPH21_1

Table S2A.

N,N′ Dimethylsphingosine (DMS) 6djz S1R (first docking run).

Clus -ter Rank	Lowest Binding Energy	Run	Mean Binding Energy	Num in Clus	Histogram 5 10 15 20 25 30 35
1	-9.43	16	-8.56	15	###############
2	-9.39	13	-8.56	6	######
3	-8.46	8	-8.46	1	#
4	-7.98	11	-7.27	7	#######
5	-4.45	18	-4.45	1	#

```
MODEL       16
USER    Run = 16
USER    Cluster Rank = 1
USER    Number of conformations in this cluster = 15
USER
USER    RMSD from reference structure       = 1.776 A
USER
USER    Estimated Free Energy of Binding    =   -9.43 kcal/mol  [=(1)+(2)+(3)-(4)]
USER    Estimated Inhibition Constant, Ki   =  122.41 nM (nanomolar)   [Temperature = 298.15 K]
USER
USER    (1) Final Intermolecular Energy     =  -12.31 kcal/mol
USER        vdW + Hbond + desolv Energy     =  -10.44 kcal/mol
USER        Electrostatic Energy            =   -1.87 kcal/mol
USER    (2) Final Total Internal Energy     =   -3.31 kcal/mol
USER    (3) Torsional Free Energy           =   +5.37 kcal/mol
USER    (4) Unbound System's Energy         =   -0.81 kcal/mol
USER
USER
USER
USER    DPF = DMS1_molA_6djz_recep_min.dpf
USER    NEWDPF move    DMS1.pdbqt
USER    NEWDPF about   11.259200 37.279700 -35.935300
```

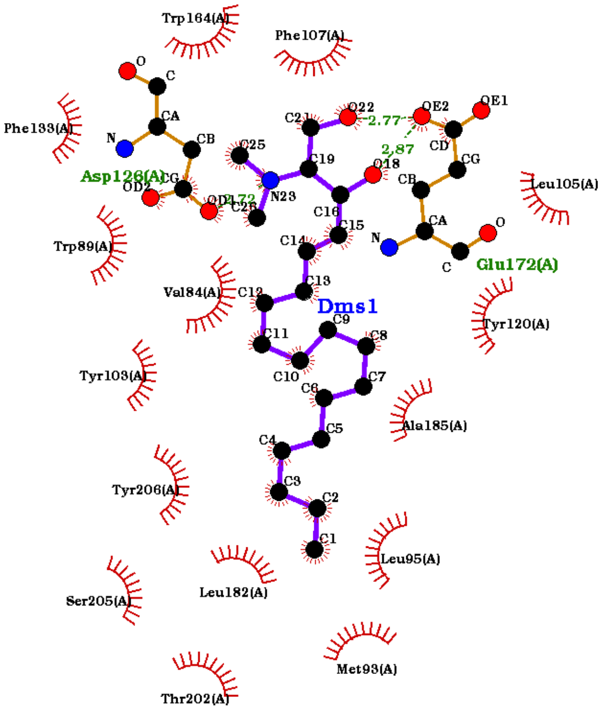

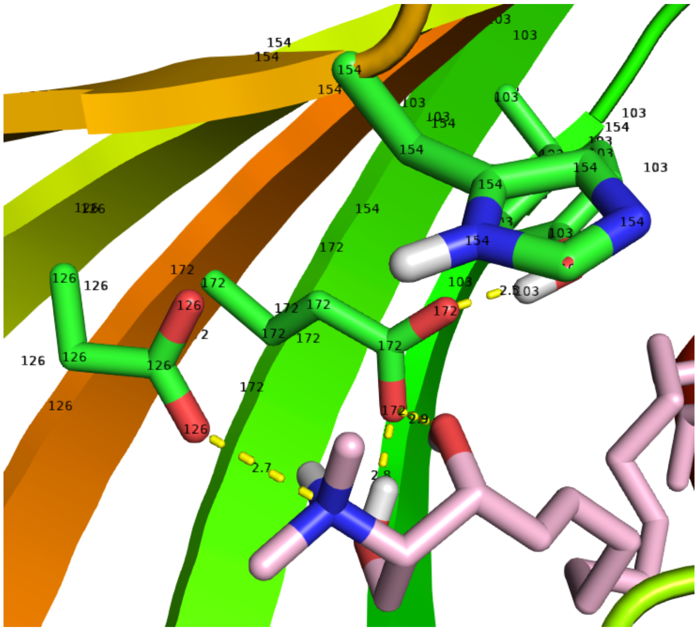

molA-6djz-recep-min_DMS1_1

Table S2B.

N,N Dimethylsphingosine (DMS) 6djz S1R (second docking run).

Clus -ter Rank	Lowest Binding Energy	Run	Mean Binding Energy	Num in Clus	Histogram (5 10 15 20 25 30 35)
1	-9.43	14	-8.15	21	#####################
2	-8.23	27	-7.97	2	##
3	-7.81	4	-7.10	4	####
4	-7.39	13	-7.39	1	#
5	-6.88	29	-6.88	1	#
6	-4.69	22	-4.69	1	#

```
MODEL       14
USER    Run = 14
USER    Cluster Rank = 1
USER    Number of conformations in this cluster = 21
USER
USER    RMSD from reference structure       = 1.756 A
USER
USER    Estimated Free Energy of Binding    =   -9.43 kcal/mol  [=(1)+(2)+(3)-(4)]
USER    Estimated Inhibition Constant, Ki   =  122.88 nM (nanomolar)   [Temperature = 298.15 K]
USER
USER    (1) Final Intermolecular Energy     =  -13.53 kcal/mol
USER        vdW + Hbond + desolv Energy     =  -11.46 kcal/mol
USER        Electrostatic Energy            =   -2.07 kcal/mol
USER    (2) Final Total Internal Energy     =   -2.07 kcal/mol
USER    (3) Torsional Free Energy           =   +5.37 kcal/mol
USER    (4) Unbound System's Energy         =   -0.81 kcal/mol
USER
USER
USER
USER    DPF = DMS2_molA_6djz_recep_min.dpf
USER    NEWDPF move    DMS2.pdbqt
USER    NEWDPF about   11.555000 37.443300 -36.209500
USER    NEWDPF tran0   11.567927 34.841621 -36.325919
```

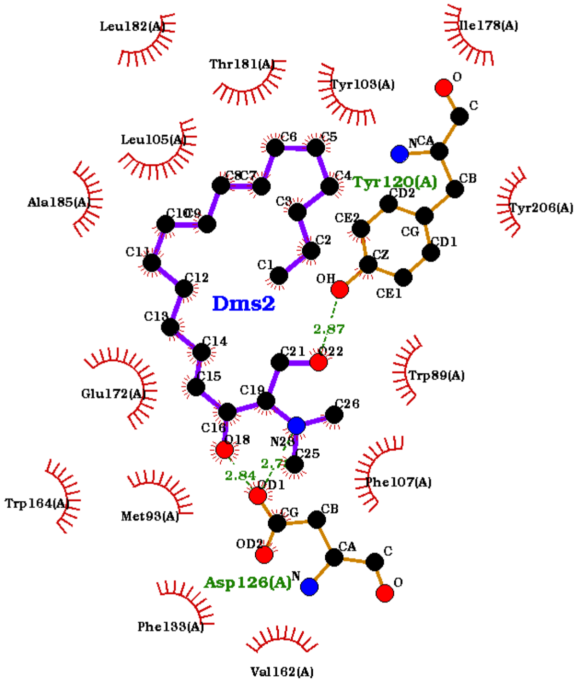

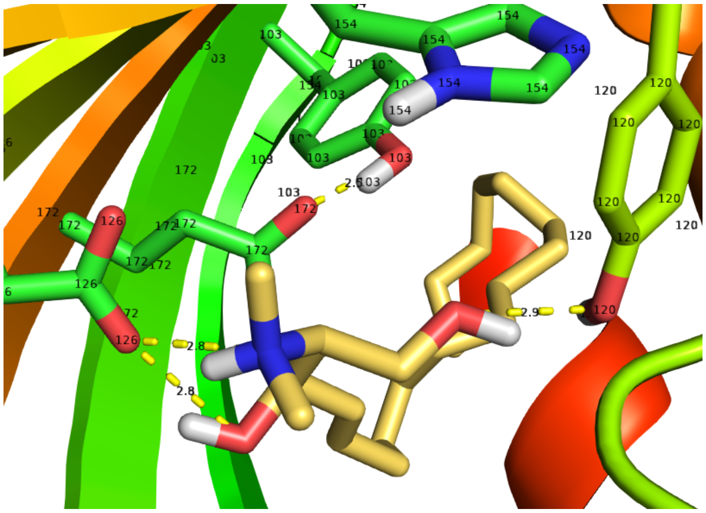

molA-6djz-recep-min_DMS2_1

SUPPLEMENTAL FIGURE-1

A.

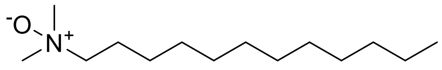

Lauryldimethylamine oxide

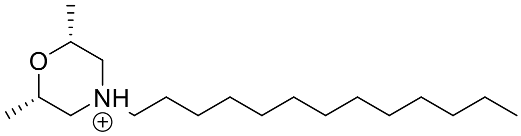

tridemorph

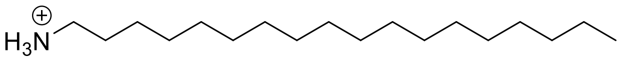

stearylamine

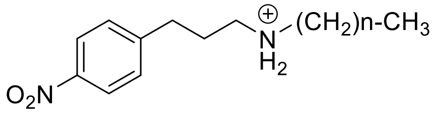

n = 3, N-3-(4-nitrophenylpropyl)tetrane-1-amine

n = 6, N-3-(4-nitrophenylpropyl)heptane-1-amine

n = 11, N-3-(4-nitrophenylpropyl)dodecane-1-amine

n = 17, N-3-(4-nitrophenylpropyl)octadecane-1-amine

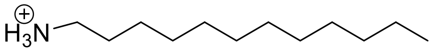

dodecylamine

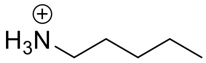

pentylamine

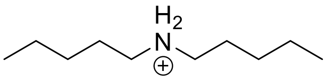

dipentylamine

B.

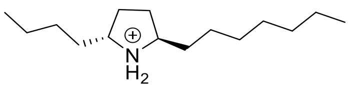

(2R-trans)-2-butyl-5-heptyl-pyrrolidine

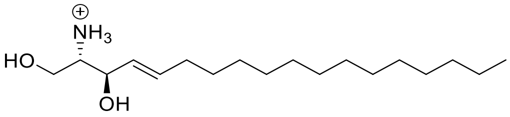

D-erythrosphingosine

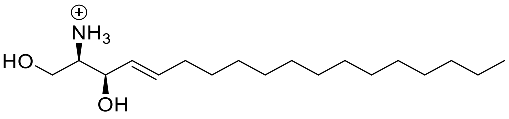

L-threosphingosine

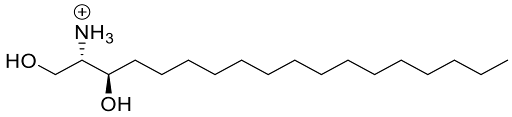

D-erythrosphinganine

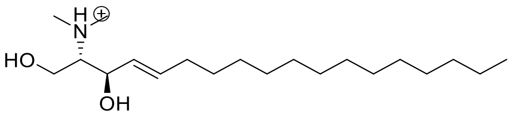

D-erythro-N,N Dimethylsphingosine

Figure S1. Exogenous and endogenous amines that bind to the S1R.

A. Exogenous amines including N-substituted compounds such as lauryldimethylamine oxide (LDAO) [Ramachandran, S., Doctoral Dissertation, UW Madison, 2008], tridemorph [51,110], dodecylamine, stearylamine [51, 55], pentylamine, dipentylamine [51] and similar compounds [52, 53] plus a series of N-3-(4-nitrophenylpropyl)-alkyl-1-amines [51, 54].

B. An endogenous amine and SPH derivatives that bind to the S1R include (2R-trans)-2-butyl-5-heptylpyrolidine [111], (SPH), L-threosphingosine, D-erythrosphinganine and D-erythro-N,N-dimethylsphingosine (DMS) [55]. With a pKa between 8-9.5 these compounds will generally be positively charged at neutral pH as ammonium salts but the charge will depend on the intracellular environment of the cell and the lipid bilayer.
